# Treatment of stiff thoracic scoliosis by thoracoscopic anterior release combined with posterior instrumentation and fusion

**DOI:** 10.1186/1749-799X-2-16

**Published:** 2007-10-15

**Authors:** Kenneth MC Cheung, Jing-ping Wu, Qing-he Cheng, Bonnie SC Ma, Ji-chang Gao, Keith DK Luk

**Affiliations:** 1 Department of Orthopedics and Traumatology, Queen Mary Hospital, The University of Hong Kong, 102 Pokfulam Road, Hong Kong, China; 2 Department of Orthopaedics, Jinshan Hospital, Fudan University, Shanghai, China; 3 The 211^th ^Hospital of PLA, Harbin, China

## Abstract

**Background:**

Thoracoscopic anterior release has been shown that it can effectively improve spinal flexibility in animal and human cadaveric studies, and has been advocated for use in patients with scoliosis. This prospective case series aims to investigate the improvement of the spinal flexibility and the effectiveness in deformity correction by anterior thoracoscopic release and posterior spinal fusion.

**Methods:**

Eleven patients with stiff idiopathic thoracic scoliosis underwent anterior thoracoscopic release followed by posterior instrumentation. The average number of discs excised was five. Spinal flexibility was assessed by the fulcrum bending technique. Cobb angle before and after the anterior release was compared.

**Results:**

The patients were followed for an average of 5.6 years (range 2.2 to 8.1 years). Fulcrum bending flexibility was increased from 39% before the thoracoscopic anterior spinal release to 54% after the release. The average Cobb angle before anterior release was 74° on the standing radiograph and 45° with the fulcrum-bending radiograph. This reduced to 34° on the fulcrum-bending radiograph after the release, and highly corresponded to the 31° measured at the post-operative standing radiograph.

**Conclusion:**

It was demonstrated in patients with stiff idiopathic thoracic scoliosis that thoracoscopic anterior spinal release can effectively improve the spinal flexibility and increase the correction of the spinal deformity.

## Background

Anterior spinal release can improve spinal flexibility and maximize correction of spinal deformity effectively when treating stiff thoracic scoliosis. It is inevitable to incise the chest wall muscles to remove intervertebral disc in the open chest procedures, which leads to multiple surgical complications such as reduced airway flow, post-operative lung collapse, blood loss, chest wall scarring and prolonged hospitalization. Nevertheless, utilizing video-assisted thoracoscopy in anterior spinal release can effectively reduce or prevent these surgical complications [1,2].

## Methodology

**Figure 1:**
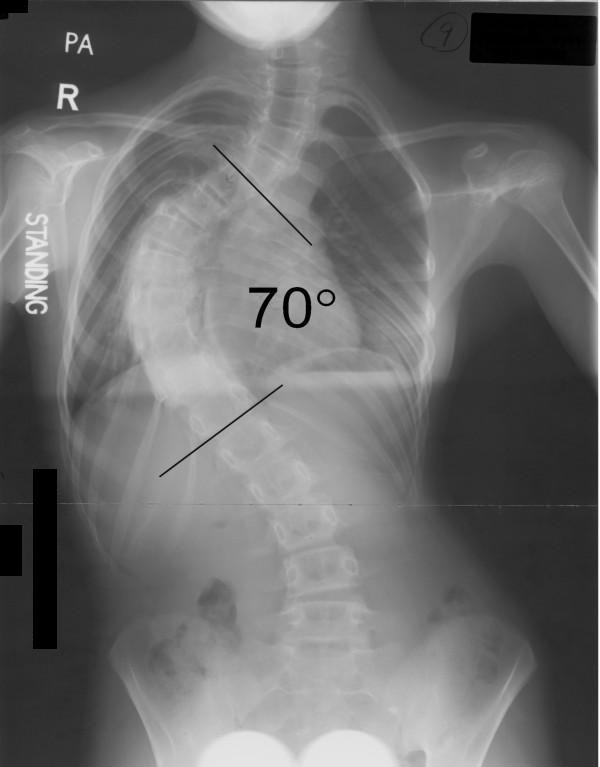
Standing radiograph before anterior release.

**Figure 2:**
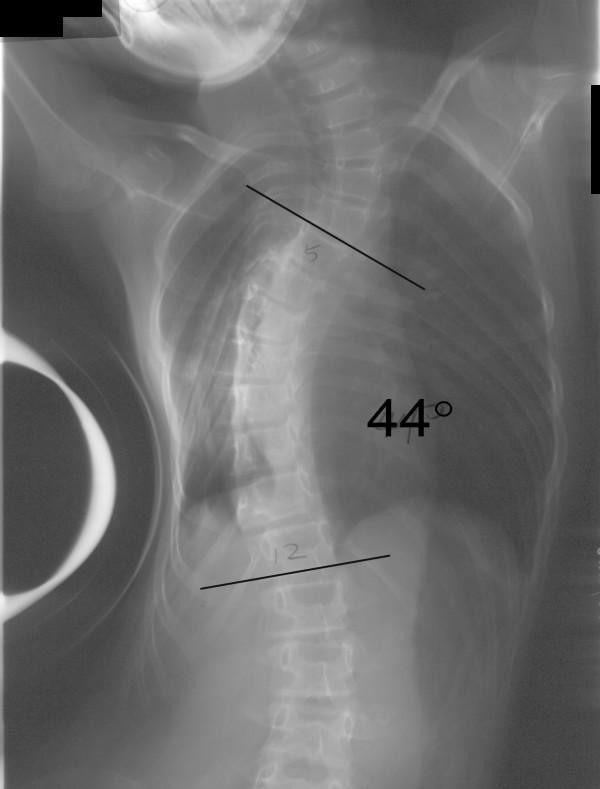
Fulcrum bending radiograph before anterior release.

**Figure 3:**
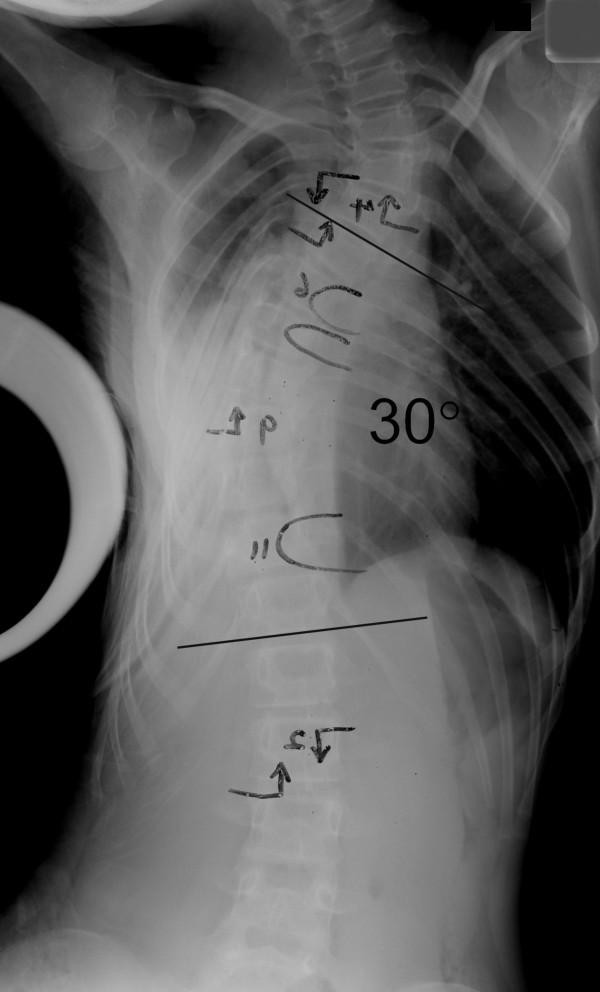
Fulcrum bending radiograph after anterior release.

Between June 1997 and June 2003, 11 patients with stiff thoracic scoliosis underwent thoracoscopic anterior release, followed by either staged (one week apart) or synchronous posterior instrumentation and spinal fusion. Routine standing anterior-posterior radiograph was taken for each patient to determine the Cobb angle (Figure 1). Definition of stiff scoliosis is that the Cobb angle being larger than 40 degrees in a fulcrum bending X-ray. The fulcrum bending radiograph was taken with a cylindrical fulcrum placed over the apex of the scoliotic curve (Figure 2, 3) [3]. The patient was asked to lie sideways over a fulcrum made from a large plastic cylinder with extra padding for comfort, using the body weight of the head and lower limbs to straighten the spine over the apex of the convex curve. The mean age at the time of operation was 16.5 years (range 11.9 – 35.5 years). According to King's classification, the curve types were as follows: type I (1), type II (5), type III (5).

**Figure 4:**
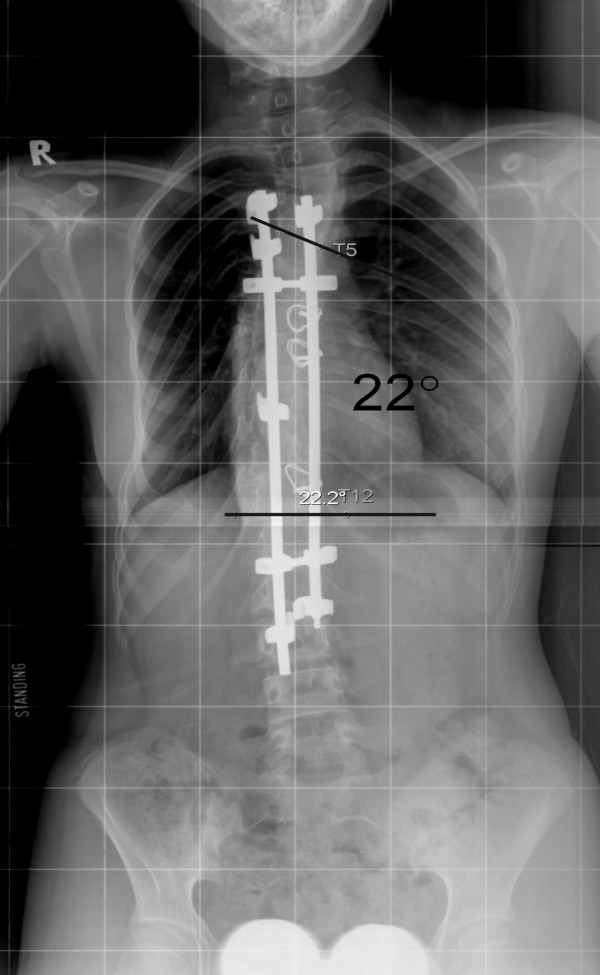
Standing radiograph after posterior instrumentation.

**Figure 5:**
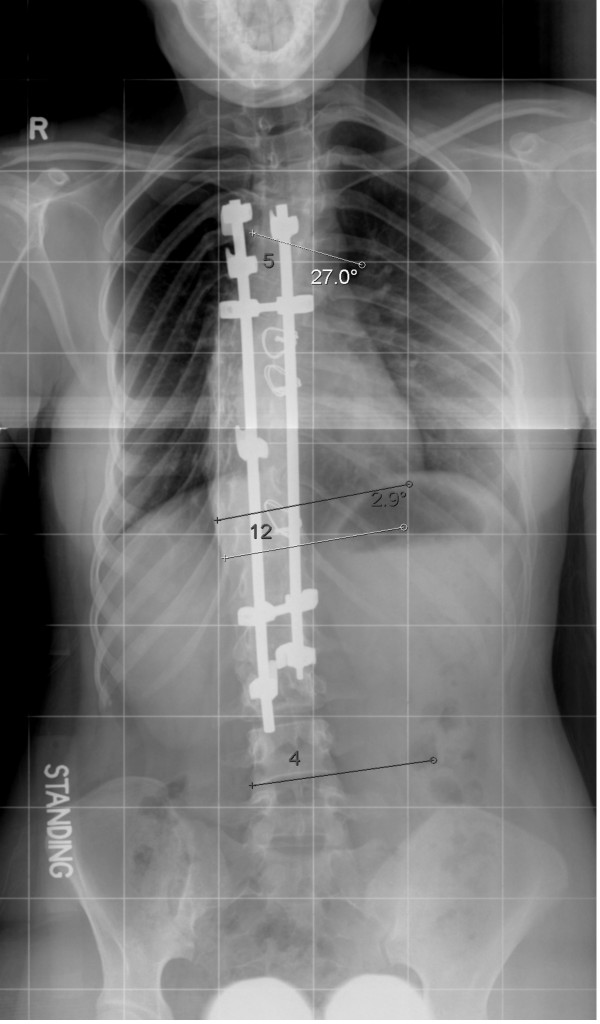
Standing radiograph 5 years after surgery.

The surgical technique of thoracoscopic-assisted anterior release was as described in the previous publication of Luk et al. [2]. In brief, the thoracoscopic anterior release was done under general anesthesia, a keyhole of 2 cm diameter was opened over the mid-axillary line at the sixth or seventh intercostal space at the convex side of the scoliotic curve. 3 to 4 more manipulative keyholes were opened near the mid-axillary line depending levels needed to be exposed. Ribs were not removed. Intervertebral discs near the apex were excised, including nucleus pulposus and cartilaginous end-plates. The posterior longitudinal ligament could be reached when excising the cephalic intervertebral disc. In general, 3–6 intervertebral discs had been excised and the average number of discs excised was five. Posterior surgery adopted pedicle hook system, and pedicle screws in the form of hybrid constructs were also used in the later part of the study (Figure 4 &5, Table 1). For King type I (double curve, lumbar major), both thoracic and lumbar curves were corrected and fused, while for King type II and type III single thoracic curves, they were selectively fused to the lower thoracic or upper lumbar spine.

**Table 1:** Number of patients with different instrumentations

Instrumentation System	Number of patients	Hooks only or hybrids
Isola	3	Hybrids
CD Horizon	4	Hook (1), Hybrids (3)
TSRH	1	Hook
Moss Miami	3	Hook (1), Hybrids (2)

Two methods were utilized to test for the effectiveness of thoracoscopic anterior release in increasing spinal flexibility. First one was the direct comparison between the pre- and post-operative angles in fulcrum bending radiographs. Second one was comparing the pre-operative fulcrum bending radiograph with the post-operative correction, using the fulcrum bending radiograph to assess the spinal fulcrum bending flexibility. The fulcrum bending flexibility was calculated as: Fulcrum Bending Flexibility (%) = (Pre-operative Cobb Angle – Fulcrum Bending Cobb Angle)/Pre-operative Cobb Angle × 100%. This fulcrum bending flexibility can be used to assess the change in spinal flexibility after anterior release. Statistical analyses were performed using paired *t*-test, with p < 0.05 being statistically significant.

## Results

All thoracoscopic anterior release surgeries were successfully done, none of the case was obliged to become open surgery. Average time for anterior release was 4.3 hours (range from 3 – 6 hours), and average blood loss was 180 ml (range from 40 – 400 ml). No obvious intra- or post-operative complications. Mean follow-up length was 5.6 years (2.2 – 8.1 years). Pre-operative mean fulcrum bending flexibility was 39%, with a statistical significant increment (p < 0.05) of 15% after anterior release, the mean fulcrum bending flexibility reached 54% post-operatively. The mean Cobb angle in standing radiograph was 74 degree before anterior release, and that in fulcrum bending radiograph was 45 degree. After anterior release, however, the mean Cobb angle in fulcrum bending radiograph was 34 degree. The actual mean Cobb angle in standing radiograph after posterior instrumentation with bone grafting was 31 degree (see table 2).

**Table 2:** Cobb angle measurements of all cases (°)

	Case 1	Case 2	Case 3	Case 4	Case 5	Case 6	Case 7	Case 8	Case 9	Case 10	Case 11	Mean
Pre-operative Standing View	65	76	75	78	61	82	80	70	70	76	75	74
Pre-operative Fulcrum Bending View	43	41	45	45	40	46	45	53	44	45	51	45
Post-operative Fulcrum Bending View	35	28	40	30	N/A	34	32	36	30	34	40	34^Δ^
Post-operative Standing View	32	28	30	28	26	29	28	33	22	32	35	31*
Correction Rate (%)	50.7	63.2	60	64.1	57.4	64.6	65	52.9	68.6	57.9	57.1	58.1
Fulcrum Flexibility (%)	46.2	63.2	46.7	61.5	N/A	58.5	60	48.6	57.1	55.3	50	57.1
FBCI (%)	109	100	128	104	N/A	110	108	109	120	105	114	102

Note 1: Compare with pre-operative fulcrum bending view ^Δ^*P *< 0.05, * *P *< 0.05

Note 2: Unable to take post-operative fulcrum bending radiograph for case 5 due to post-operative wound pain

## Discussion

Open chest surgery was adopted to improve the spinal flexibility in stiff thoracic scoliosis. With the aid of video-assisted thoracoscopic surgery (VATS), the traditional open chest anterior release surgery could be replaced with micro-trauma and less complications [4-6]. While some physicians thought thoracoscopic release and open chest release have different abilities to release the spine, and that only open chest surgery excising ribs and complete excision of intervertebral discs could successfully and completely release a stiff spine. VATS has been shown that it can effectively improve spinal flexibility in animal and human cadaveric studies [7]. Its use in human patients with scoliosis have been supported by a number of studies [8-10], however, all except one case report demonstrated that it was effective at improving spinal flexibility.

In our study, stiff scoliosis curve was defined as the residual Cobb angle equal to or larger than 40 degrees in a fulcrum bending radiograph. This concept of fulcrum bending flexibility was first suggested and applied clinically by the authors. It was used to select the fusion segments and predict the correction after surgery, so that patients and their family could be informed the treatment effect pre-operatively [11-14]. Previous researches showed that this can reflect the spinal flexibility, comparison between pre-operative fulcrum bending radiographs and post-operative correction demonstrated 98% accuracy, and the fulcrum bending flexibility can predict the post-operative correction of rib hump accurately [13]. The fulcrum bending flexibility applied in this study served as an excellent method to judge the treatment effect of thoracoscopic anterior release. This cohort included 11 patients with stiff thoracic scoliosis, with pre-operative fulcrum bending Cobb angle larger than 40 degrees, and a mean of 45 degrees. The mean post-thoracoscopic fulcrum bending Cobb angle was 34 degrees, while the mean Cobb angle in standing radiograph after posterior instrumentation was 31 degrees. These two were so close, and it demonstrated that the pre-operative fulcrum bending flexibility could accurately predict the result of surgical correction. The side-bending radiographs taken in supine lying could roughly predict the post-operatively correction, thus the fulcrum bending radiograph is superior to the traditional side-bending radiograph to predict the pos-operative Cobb angle.

Fulcrum bending flexibility is expressed as the difference between the Cobb angles measured on the fulcrum bending and preoperative radiographs divided by the preoperative Cobb angle [13]. In this series, the mean fulcrum bending flexibility also improved from 39% pre-operatively to 54% post-operatively, with 15% increment. This is a strong evidence for thoracoscopic anterior release could improve spinal flexibility among patients with stiff thoracic scoliosis, so that the curves could be corrected.

When describing surgical correction, the authors would propose that the spinal flexibility need to be taken into account, and that this is best decribed by the fulcrum bending correction index (FBCI). The FBCI is expressed as correction rate divided by fulcrum flexibility; an FBCI of 100% indicates that the surgical correction has taken up all the flexibility as revealed by the fulcrum bending radiograph [13]. In this cohort, all patients had a FBCI of larger than 100%, meaning anterior thoracoscopic release with posterior spinal fusion could over-correct the stiff scoliosis curves. In fact, previous study by the authors demonstrated that ability to correct scoliosis deformity using four different instrumentations was the same [15].

Due to inexperience at the beginning, only 3–4 intervertebral discs were excised during thoracoscopic anterior release, and the posterior surgery was performed 1 week later. It did, however, provide the conditions to prove the effectiveness of anterior release, and verify the in vivo spinal flexibility improvement with anterior release. With increasing experience, 5–6 intervertebral discs were excised in recent cases, and posterior instrumentation with bone grafting could be done at the same stage. Anterior release excised 5 intervertebral discs on average, with mean improvement of 16 degrees, illustrated that excision of a intervertebral disc could correct approximately 3 degrees. Although after excision of intervertebral discs, no anterior bone grafting was performed, the follow-up radiographs after 3 years revealed good fusion status, which showed that solely posterior bone grafting could achieve satisfactory fusion.

## Competing interests

The author(s) declare that they have no competing interests.

## Authors' contributions

KMCC participated in the design of the study and carried out the thoracoscopic surgery. JPW assessed the radiographic Cobb's angles and assisted in the surgery. QHC performed the statistical analysis and helped to draft the Chinese manuscript. BSCM drafted the manuscript. JCG collected the data. KDKL conceived of the study, and participated in the design of the study and coordination. All authors read and approved the final manuscript.
